# Cd and Zn interactions and toxicity in ectomycorrhizal basidiomycetes in axenic culture

**DOI:** 10.7717/peerj.4478

**Published:** 2018-03-07

**Authors:** Vinicius H. De Oliveira, Mark Tibbett

**Affiliations:** Centre for Agri-Environmental Research & Soil Research Centre, School of Agriculture, Policy and Development, University of Reading, Reading, Berkshire, United Kingdom

**Keywords:** Heavy metal toxicity, Metal interaction, Ectomycorrhizal fungi, Toxicity threshold

## Abstract

**Background:**

Metal contamination in soils affects both above- and belowground communities, including soil microorganisms. Ectomycorrhizal (ECM) fungi are an important component in belowground community and tolerant strains have great potential in enhancing plant-based remediation techniques. We assessed cadmium and zinc toxicity in five ECM species in liquid media (*Hebeloma subsaponaceum*; *H. cylindrosporum*; *H. crustuliniforme*; *Scleroderma* sp.; *Austroboletus occidentalis*) and investigated the potential of Zn to alleviate Cd toxicity. Due to highly divergent results reported in the literature, liquid and solid media were compared experimentally for the first time in terms of differential toxicity thresholds in Cd and Zn interactions.

**Methods:**

A wide range of Cd and Zn concentrations were applied to ectomycorrhizal fungi in axenic cultures (in mg L^−1^): 0; 1; 3; 9; 27; 81; 243 for the Cd treatments, and 0; 1; 30; 90; 270; 810; 2,430 for Zn. Combined Zn and Cd treatments were also applied to *H. subsaponaceum* and *Scleroderma* sp. Dry weight was recorded after 30 days, and in case of solid medium treatments, radial growth was also measured.

**Results and Discussion:**

All species were adversely affected by high levels of Cd and Zn, and *A. occidentalis* was the most sensitive, with considerable biomass decrease at 1 mg L^−1^ Cd, while *Scleroderma* sp. and *H. subsaponaceum* were the most tolerant, which are species commonly found in highly contaminated sites. Cd was generally 10 times more toxic than Zn, which may explain why Zn had little impact in alleviating Cd effects. In some cases, Cd and Zn interactions led to a synergistic toxicity, depending on the concentrations applied and type of media used. Increased tolerance patterns were detected in fungi grown in solid medium and may be the cause of divergent toxicity thresholds found in the literature. Furthermore, solid medium allows measuring radial growth/mycelial density as endpoints which are informative and in this case appeared be related to the high tolerance indices found in *H. subsaponaceum*.

## Introduction

Cadmium (Cd) is one of the most hazardous metals in the environment, ranked seventh in toxicity by the Agency for Toxic Substance and Disease Registry (ATSDR, 2017), it lacks any known biological function, it can be toxic to living organisms at relatively low concentrations (Alloway, 2013) and has a high mobility in soils (Lei et al., 2010). Cd can be frequently found in zinc (Zn) bearing minerals (Alloway, 2013) and due to their similar geochemical characteristics they are often associated in soils (Kabata-Pendias & Pendias, 2001). Although Zn is a micronutrient, high concentrations in the environment can be extremely harmful to biota. Data suggest that Zn can be more toxic to soil organisms than Pb (Ross & Kaye, 1994) and decrease bacterial diversity in contaminated lands (Moffett et  al., 2003).

In metal contaminated soils, symbiotic fungi such as ectomycorrhizal fungi (ECM) may improve plant fitness and metal tolerance, such as by promoting better growth or nutrition, preventing metal uptake and protecting against other abiotic and biotic stresses (Krznaric et al., 2009; Rodriguez & Redman, 2008; Zheng, Fei & Huang, 2009), being crucial for plant survival in such environments (Saraswat & Rai, 2011). Almost all land plants depend on symbiotic mycorrhizal fungi (Leyval, Turnau & Haselwandter, 1997), with woody pioneers species relying mostly on phenotypic plasticity and ectomycorrhizal assossiations to withstand metal-polluted soils (Colpaert, 2008; Krpata et al., 2008). However, the extent of the ameliorating effects of the symbiosis is difficult to demonstrate and depends on the fungal species, plant genotype (Krznaric et al., 2009) and the differential toxicity of metals (Fomina et al., 2005).

Several studies focus on assessing metal toxicity in different ECM fungi *in vitro* in order to identify tolerant species and strains (Fomina et al., 2005; Blaudez et al., 2000) , but comparisons are difficult when the variety of methods employed, with different fungi strains, range of metal concentrations and endpoints considered (e.g., radial growth or biomass production). The types of media used can also vary in results, as well as their physical states: liquid or solid agar (Colpaert et al., 2004; Tam, 1995; Zheng, Fei & Huang, 2009), which appears to be responsible for a variation in bioavailability and therefore cause a distinct difference in the toxicity thresholds for Cd and Zn (Table 1). Interactions between metals are also responsible for variation in toxicity responses; for instance, in some cases it has been observed that Zn is able to reduce Cd toxicity in certain ECM fungi, often attributed to the ionic competition for binding sites (Hartley et al., 1997).

**Table 1:** Reports on Cd and Zn toxicity thresholds in Ectomycorrhizal fungi in solid and liquid media. Toxicity thresholds for Cd and Zn in ectomycorrhizal fungi grown in either liquid or solid media. Toxic concentrations were considered as the minimum concentration to cause adverse effect or as the only toxicity value reported by the author(s).

	Toxic concentrations (mg L^−1^)	
	Solid	Liquid
Zn		
*mean*	309	123
*median*	292	22
*maximum*	975	500
Cd		
*mean*	12	2.2
*median*	2.0	0.9
*maximum*	50	10
ECM species tested	17	12
References consulted	11^a^	5^b^

**Notes.**

a Blaudez et al., 2000b; Brown & Wilkins, 1985; Colpaert & Van Assche, 1987; Colpaert & Van Assche, 1992; Colpaert et al., 2000; Colpaert et al., 2004; Colpaert et al., 2005; Denny & Wilkins, 1987; Krznaric et al., 2009; Ray et al., 2005; Willenborg, Schmitz & Lelley, 1990.

b Colpaert & Van Assche, 1987; Courbot et al., 2004; Grazzioti et al., 2001; Hartley, Cairney & Meharg, 1997; Tam, 1995.

Given the ambiguities across published dataset, we aimed to elucidate our current understanding of metal toxicity by addressing specific issues such as: the possible Zn and Cd antagonistic/synergistic interactions in ectomycorrhizal fungi, the ability of Zn in alleviating Cd toxicity effects; and the different toxicity thresholds arising from using either liquid or solid media under the same range of concentrations.

## Materials and Methods

### Assessing Cd and Zn toxicity

Toxicity trials were performed *in vitro* using five ECM species originated from non-polluted environments: *Hebeloma subsaponaceum* (from a Boreal Forest, Norway); *H. cylindrosporum* (from under pine trees, France); *H. crustuliniforme* (from Sitka spruce, Brown Earth); *Scleroderma* sp. (woodlands, Western Australia) and *Austroboletus occidentalis* (Western Australia), a species recently found to be a non-colonizing fungal partner (Kariman et al., 2014). These species were selected from our in-house collection due to their growth rates observed previously in agar medium. Methods were based on a previous study by Chen & Tibbett (2007). Four circular plugs (1 mm) were cut out from the edges of actively growing colonies (five weeks old) and transferred to Petri dishes with 25 ml of Melin-Norkrans liquid medium (MMN). The medium composition was: 6.51 mM NH_4_NO_3_, 0.57 mM MgSO_4_ ⋅ 7H_2_O, 0.23 mM CaCl_2_, 0.015 mM ZnSO_4_, 0.3 mM Thiamine, 5.55 mM d-glucose, 2 mM KH_2_PO_4_, 0.035 mM Ferric EDTA; pH was adjusted to 5.5. No Zn (ZnSO_4_) was added to the initial MMN medium used for the Zn treatments, as this metal was added later to make up the desired range of concentrations.

Cd and Zn concentrations were added via CdCl_2_ and ZnSO_4_ solutions to the final medium, and the final concentrations were (in mg L^−1^): 0; 1; 3; 9; 27; 81; 243 for the Cd treatments, and 0; 1; 30; 90; 270; 810; 2,430 for the Zn treatments. Such concentrations were selected based on similar toxicity experiments with mycorrhizal fungi found in the literature (Blaudez et al., 2000; Colpaert & Van Assche, 1992; Colpaert et al., 2004; Ray et al., 2005; Tam, 1995; Willenborg, Schmitz & Lelley, 1990).

The fungal cultures were incubated in the dark at 20 °C for 30 days, each treatment had four replicates. The mycelial mats were then removed from the medium, placed on small aluminum envelopes (weighed previously) and oven-dried overnight at 60 °C. The dry weight (DW) was assessed gravimetrically. The Tolerance Index (TI%) was used to express the tolerance results (Fomina et al., 2005), calculated by the equation:

(1) \begin{eqnarray*}TI(\text{%})= \frac{DWtreated}{DWcontrol} \times 100.\end{eqnarray*}

In which DW is the dry weight obtained from the fungal biomass.

Statistical analysis was performed on the dry weight data using STATISTICA 12^®^. To attain normal distribution (Shapiro–Wilk), box-cox transformation was applied. However, the data did not meet the assumption of homogeneity of variances (Levene’s test). Thus, analysis of variance was carried out using Welch’s test (Zar, 2010), followed by Dunnett’s test to determine the LOAEC values (Lowest Observed Adverse Effects Concentration), which also does not require equal variances (Quinn & Keough, 2002). The Dunnett’s for Zn toxicity considered the treatment of 1 mg L^−1^ Zn as the control.

### Cd and Zn interactions

To verify the effect of Zn in preventing Cd toxicity in ECM fungi, a second experiment was carried out using the same methods described above, except no basal Zn was added to the basic MMN medium in all treatments, and was added later to make up the desired range of concentrations; growth period of 21 days. However, because *H. cylindrosporum* had lower or similar performance as *H. subsaponaceum*, the former was excluded from this experiment. In this case, ECM species were exposed to Cd and Zn together, with concentrations added in different combinations: 0, 1 and 9 mg L^−1^ for Cd, and 0, 1, 9 and 30 mg L^−1^ for Zn. Therefore, this assay was comprised of 12 treatments (Cd × Zn: 0 × 0, 0 × 1, 0 × 9, 0 × 30, 1 × 0, 1 × 1, 1 × 9, 1 × 30, 9 × 0, 9 × 1, 9 × 9, 9 × 30 mg L^−1^).

Relative dry weight was calculated with Eq. (1), and ANOVA followed by Tukey’s test were performed to verify significant differences among the Zn treatments (0; 1; 9 and 30 mg L^−1^). For attaining normality and homoscedasticity in two variables (1 mg L^−1^ Cd in *H. crustuliniforme* and 0 mg L^−1^ Cd in *Scleroderma* sp.), data were transformed by the equation: 1∕*x*.

Due to the high Cd toxicity observed, this experiment was repeated subsequently with only *Scleroderma sp.* and *Hebeloma subsaponaceum*, but using another range of concentrations (0; 1; 9 mg L^−1^ Cd and 0; 30; 60; 120 mg L^−1^ Zn) and two types of MMN media, a solid medium containing 2% agar, and a liquid medium as described previously, with four replicates. Plates were incubated in the dark, at 20 ± 2 °C for 30 days. By the end of the growth period, treatments with solid media were measured for radial growth (a mean between vertical and horizontal diameters, in centimeters). After which the agar was cut and removed from the plates and melted in a microwave in short 15 s burst for no more than one minute in total (Karaduman, Atli & Yamaç, 2012); the mycelium was removed and blotted dry with absorbent paper until it was free of all agar medium, the mycelium was then washed with deionized water, oven-dried overnight (60 °C) and weighed. Liquid media treatments were handled as described previously. Statistical analyses were performed following the same steps as the previous experiments. Contour plots were achieved by linear interpolation (using SigmaPlot^®^; Systat Software Inc., San Jose, CA, USA) of the fungal Tolerance Indexes (TI%, but in this case considering 100% as the treatment with the highest biomass production: i.e., Cd × Zn (0 × 30 mg L^−1^ in liquid cultures and Cd × Zn (1 × 30 mg L^−1^) in solid cultures); using 12 Zn × Cd co-ordinates, based on publications by Hartley et al. (1997) and Krznaric et al. (2010).

## Results

**Figure 1:**
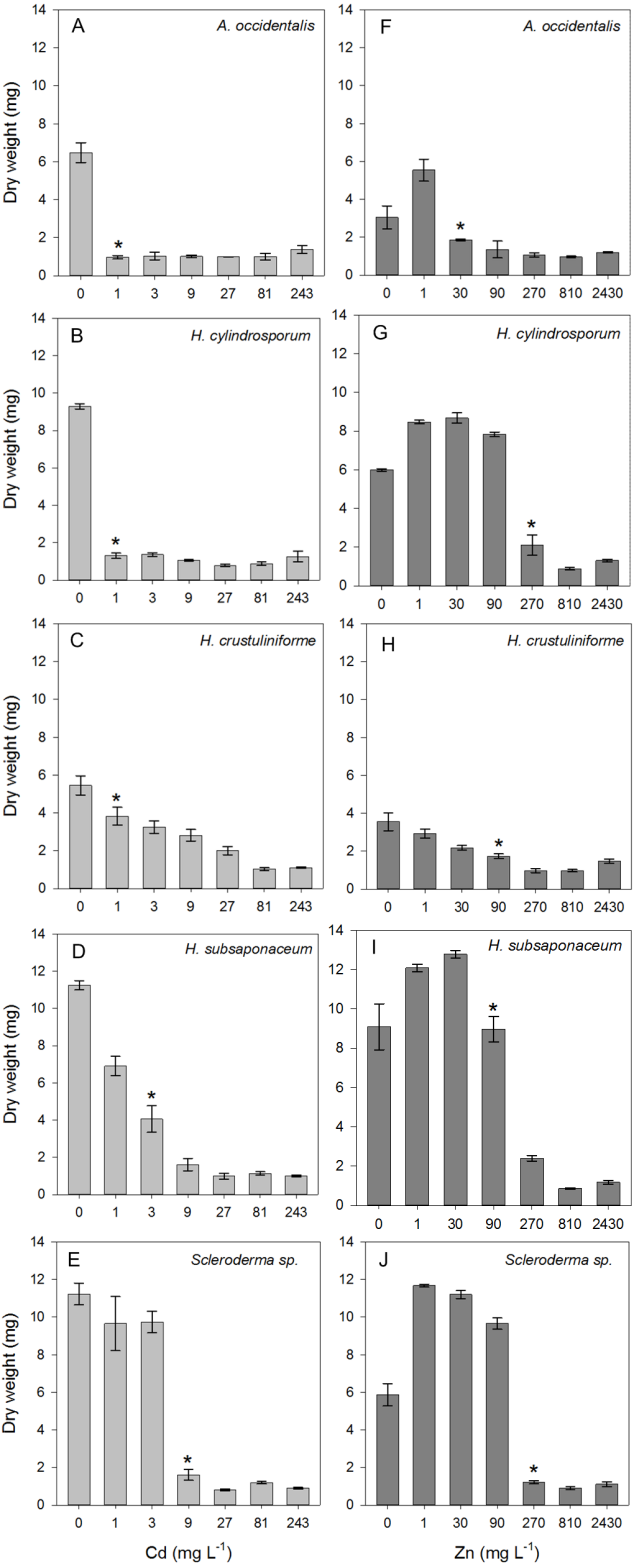
Toxicity thresholds of Cd and Zn in five ectomycorrhizal species. Dry weight of five ECM species (*Austroboletus occidentalis*, *Hebeloma cylindrosporum, H. crustuliniforme, H. subsaponaceum*, *Scleroderma* sp.) after 30 days under a range of Cd (A–E) or Zn (F–J) concentrations in liquid media. Asterisks represent the first concentration from which fungal growth starts to be adversely affected, LOAEC, determined by Dunnett’s test (*p* < 0.05). LOAEC for Cd and Zn (in mg L^−1^) were, respectively, 1 and 30 in *A. occidentalis*; 1 and 270 in *H. cylindrosporum*; 1 and 90 in *H. crustuliniforme*; 3 and 90 in *H. subsaponaceum*; 9 and 270 in *Scleroderma* sp.

All species assessed were negatively affected by either Cd or Zn, depending on the concentration they were under, although lower Zn concentrations had a positive effect on all strains (Fig. 1). Biomass decreased in all species exposed to Cd, and a critical effect was observed in *A. occidentalis*, *H. cylindrosporum* and *H. crustuliniforme* in concentration as low as 1 mg L^−1^, highlighting Cd pronounced toxicity. There was no visible growth at highest Cd and Zn concentrations, thus the dry weight detected in these cases, i.e., <2 mg (Fig. 1) were considered as being from the four circular agar plugs (1 mm) initially used for inoculation. Reduced biomass due to Cd and Zn toxicity is a common consequence observed in ECM fungi, regardless the species. Cadmium, for being an element with no known biological function, is considerably more toxic than Zn and its toxic effects began at concentrations at least 30 times lower than the concentrations necessary for Zn to display toxicity (Fig. 1). Nonetheless, Zn toxicity was observed in lower concentrations than expected, three species had LOAEC values of 90 mg L^−1^ (*H. crustuliniforme* and *H. subsaponaceum*) or lower (*A. occidentalis*) (Fig. 1). From the LOAEC values determined, the most sensitive species to metal toxicity considering both Cd and Zn, were *A. occidentalis* and *H. cylindrosporum*, while *Scleroderma* sp. and *H. subsaponaceum* were the most tolerant.

**Figure 2:**
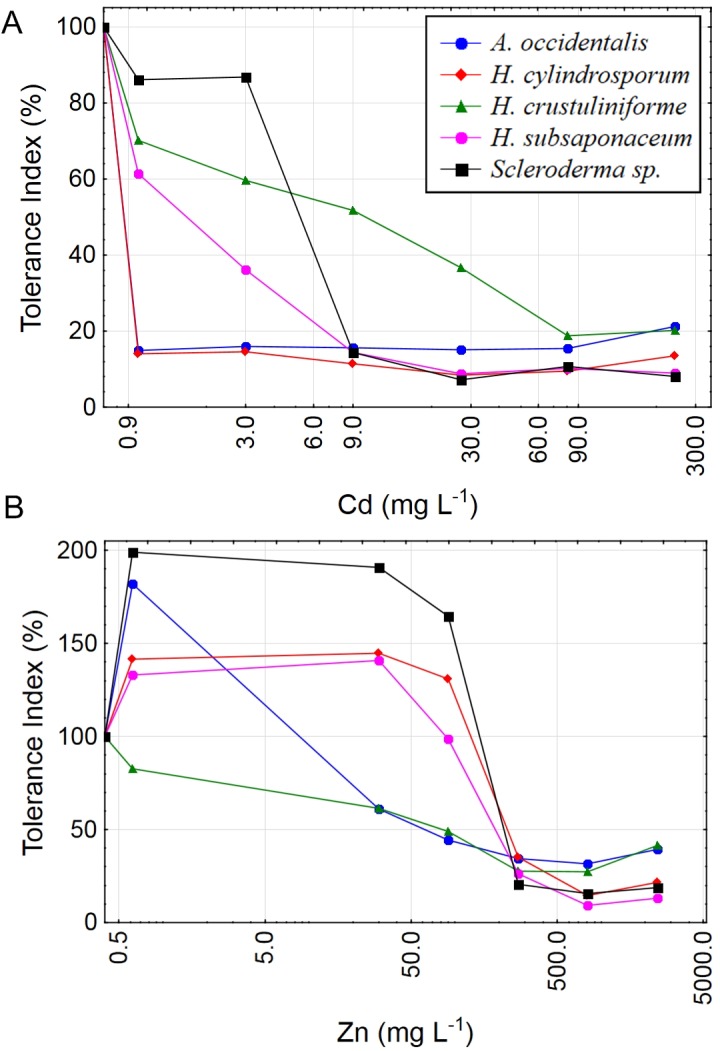
Tolerance index for five ectomycorrhizal fungi exposed to Cd and Zn. Metal tolerance indices (TI%) for five ECM species under increasing concentrations of Cd: 0; 1; 3; 9; 27; 81 and 243 mg L^−1^ (A) or Zn: 0; 1; 30; 90; 270; 810 and 2,430 mg L^−1^ (B) in liquid media. *X* axes are in logarithmic scale. *TI*% = DW treated/DW control × 100.

Almost all species had higher growth under low concentrations of Zn, except for *H. crustuliniforme*, which was the only species not to show any growth improvement even at the lowest Zn treatment, of 1 mg L^−1^ (Fig. 2), a concentration long considered to be beneficial and typically part of the basic formulation of fungal growth media (Marx & Bryan, 1975; Pridham & Gottlieb, 1948; Tibbett et al., 1999).

**Figure 3:**
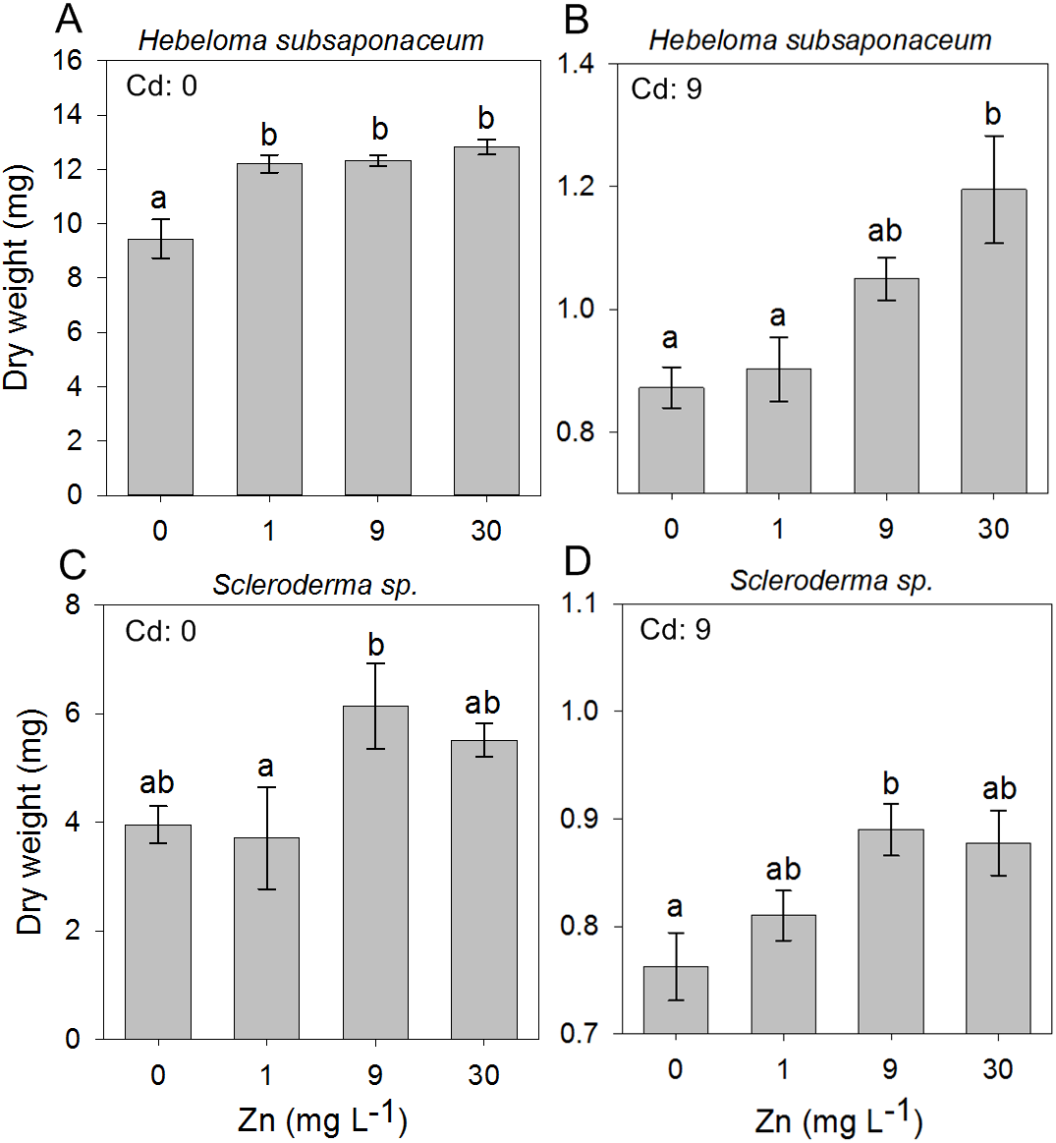
Effect of Zn addition on ectomycorrhizal cultures exposed to Cd. Effects of Zn concentrations on dry weights (mean, *n* = 4) of *Hebeloma subsaponaceum* (A–B) and *Scleroderma* sp. (C–D) under two Cd concentrations (0 and 9 mg L^−1^). Data for other species were not significantly different and therefore are not shown. Different letters represent significant differences by Tukey test (*p* < 0.05).

In the second experiment, in which the ECM fungi were exposed to mixed concentrations of Cd and Zn, it was observed that Zn addition had little effect on the dry weight of all species, regardless of the Cd concentration, except for *Scleroderma* sp. and *H. subsaponaceum*: the only species in which Zn addition promoted biomass increase at both non-contaminated media (Cd: 0 mg L^−1^) and highest Cd concentration, of 9 mg L^−1^ (Fig. 3).

Because both *A. occidentalis* and *H. crustuliniforme* had poor biomass production and suffered highly from Cd and Zn toxicity, results for these two species are not shown; based on previous results (Fig. 1), their responses were entirely predictable.

In a concluding experiment *H. subsaponaceum* and *Scleroderma* sp. were exposed to Cd along with higher Zn concentrations, in both solid and liquid media. Dry weight and radial growth (in solid media only) were evaluated (Table 2). In general, Zn addition did not alleviate Cd toxicity effects in both species, however there were a few exceptions: at 1 mg L^−1^ Cd, the addition of Zn (30 mg L^−1^) promoted a dry weight increase in *Scleroderma* sp. (from 2.7 to 10.5 mg) in liquid media. However this effect was not significant in solid media (Table 2). In *H. subsaponaceum,* 30 mg L^−1^ of Zn was beneficial at the highest Cd concentration (9 mg L^−1^), in solid media, but the same was not observed in liquid media.

**Table 2:** Cd and Zn effects on dry weight and radial growth of *Hebeloma subsaponaceum* and *Scleroderma sp.* Fungal dry weight (mg) and radial growth (cm) of *Hebeloma subsaponaceum* and *Scleroderma* sp. grown in liquid and solid media containing different Cd and Zn concentrations (mean ± SE).

		*H. subsaponaceum*			*Scleroderma* sp.		
	Zn (mg L^−1^)	Cd (mg L^−1^)					
		0	1	9	0	1	9
Liquid media dry weight (mg)	0	11.0 ± 0.1	10.4 ± 1.1	2.7 ± 0.3	4.4 ± 1.6	2.7 ± 0.8	1.6 ± 0.1
	30	21.1 ± 3.9^a^	12.0 ± 0.3	2.8 ± 0.2	9.8 ± 0.6^a^	10.5 ± 0.3^a^	1.3 ± 0.1
	60	11.5 ± 0.2	12.9 ± 0.3	3.4 ± 0.3	6.0 ± 0.4	2.0 ± 0.3	1.3 ± 0.1
	120	9.2 ± 1.1	9.1 ± 1.2	2.6 ± 0.2	1.6 ± 0.2	1.5 ± 0.1	1.1 ± 0.1
Solid media dry weight (mg)	0	10.8 ± 0.6	10.2 ± 0.8	4.5 ± 0.4	17.0 ± 2.7	16.9 ± 1.3	14.8 ± 1.3
	30	9.2 ± 0.6	10.7 ± 1.0	5.6 ± 0.1^a^	18.5 ± 1.6	19.8 ± 2.9	12.2 ± 1.5
	60	9.1 ± 0.1	8.2 ± 0.2	5.5 ± 0.1	19.4 ± 1.7	14.4 ± 0.4	12.8 ± 1.0
	120	8.2 ± 0.5	7.9 ± 0.5	4.1 ± 0.3	17.3 ± 1.0	11.7 ± 0.8^b^	7.9 ± 2.5^b^
Solid media radial growth (cm)	0	3.1 ± 0.1	2.6 ± 0.0	1.3 ± 0.1	6.0 ± 0.1	5.9 ± 0.2	4.3 ± 0.1
	30	2.8 ± 0.2	2.5 ± 0.0	1.2 ± 0.0	6.1 ± 0.2	6.5 ± 0.2	5.0 ± 0.2
	60	2.7 ± 0.1	2.4 ± 0.1^b^	1.2 ± 0.0	6.5 ± 0.1	6.4 ± 0.2	5.9 ± 0.2^a^
	120	2.4 ± 0.1^b^	2.3 ± 0.0^b^	1.0. ± 0.0^b^	6.8 ± 0.2^a^	6.3 ± 0.1	4.3 ± 0.4

**Notes.**

a Mean values higher than the control (Zn: 0 mg L^−1^) in each Cd treatment.

b Mean values lower than the control; all by Dunnett’s test (*p* < 0.05).

In a few instances, toxicity was even more acute in the presence of both Cd and Zn, such as the dry weight decrease in *Scleroderma* sp. at 120 mg L^−1^ Zn, but only in the presence of Cd, suggesting a synergistic toxicity. Similar effect was also observed in the radial growth of *H. subsaponaceum* (Table 2), in which there was a decrease in the radial growth at 120 mg L^−1^ Zn in *H. subsaponaceum* for all Cd treatments, however, the dry weight was not affected in these cases. As for *Scleroderma* sp., radial growth was not negatively affected despite either Cd or Zn additions.

**Figure 4:**
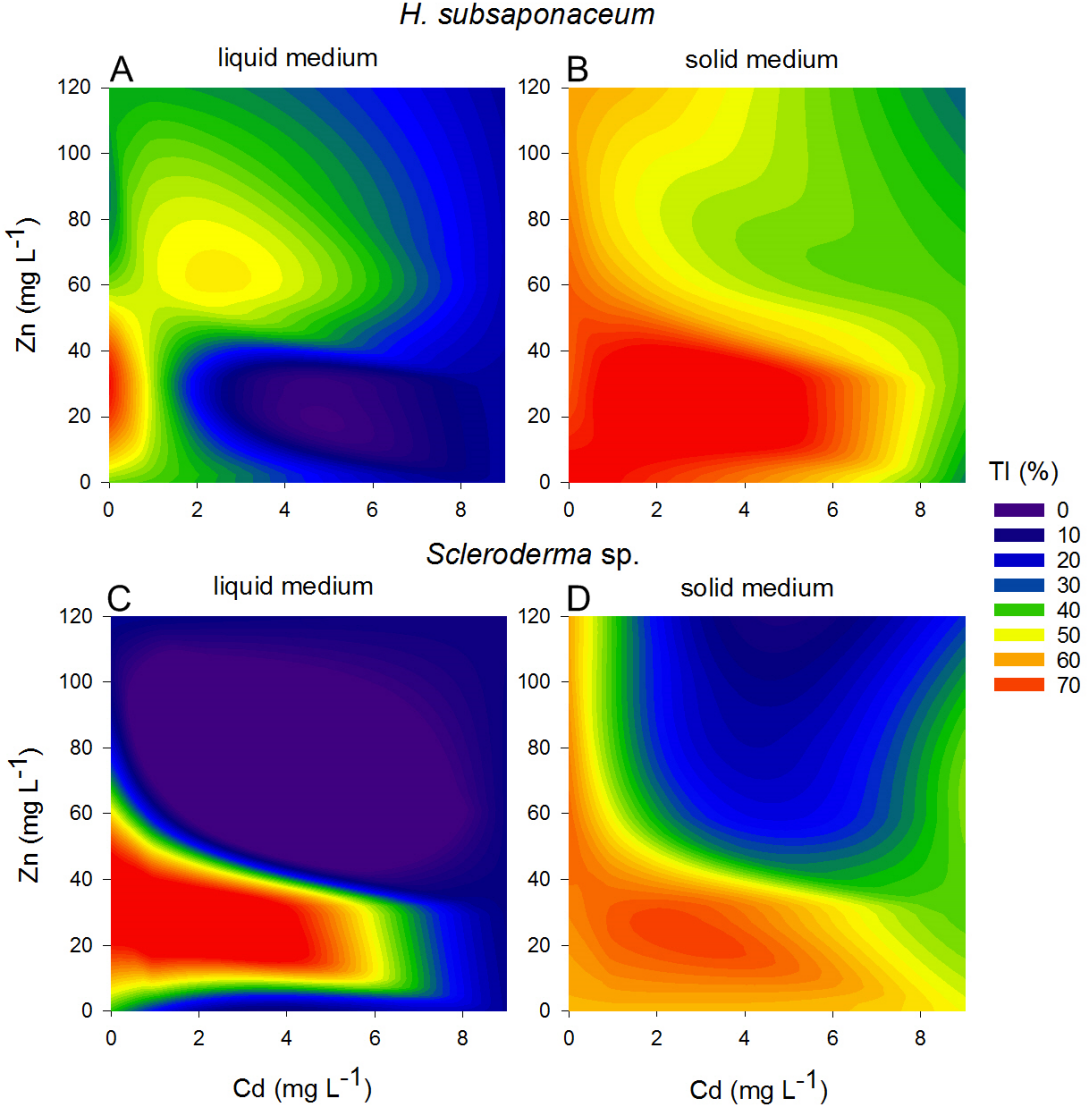
Contour plots showing different tolerance patterns of ectomycorrhizal fungi grown in solid and liquid media contaminated by Zn and Cd. Contour plots: tolerance indices (TI%) for *H.  subsaponaceum* (A–B) and *Scleroderma* sp. (C–D) exposed to Cd and Zn *in vitro* in two types of Modified Melin-Norkrans media, liquid (left) and solid (right). *TI*% = DW treated/DW control × 100. The reference value (100%) was considered as the treatment which produced the most biomass (dry weight). Contour plots produced by linear interpolation. High TI% (orange and red) are associated with lower toxicity, while low TI% (purple and blue) with higher toxicity.

Contour plots were created using the Tolerance Index of the dry weight of *Scleroderma* sp. and *H. subsaponaceum* in order to visualize the different responses between the cultures grown in solid and liquid media (Fig. 4). *Scleroderma* sp. was very sensitive to increasing Cd and Zn concentrations, but around 30 mg L^−1^ Zn it exhibited distinct tolerance (≥70%), even in the presence of 1 mg L^−1^ Cd and in both types of media. Despite this increment in the tolerance index caused by Zn, it is clear that higher Zn concentrations were extremely toxic to this species at higher Cd doses (Fig. 4). Tolerance indices were in general considerably higher in solid media, for instance, in *H. subsaponaceum* tolerance index was mostly over 50% in solid media, while in liquid media it was mainly around 40% or lower (Fig. 4).

## Discussion

Reports show that there is a great variation in Cd tolerance among ECM fungal species but generally Cd causes toxicity at around 1 mg L^−1^ *in vitro* (Colpaert & Van Assche, 1992; Ray et al., 2005; Tam, 1995). Our data is in keeping with this general tenet, which applies to a number of different genera, such as *Laccaria*, *Scleroderma*, *Suillus*, *Pisolithus, Cenococcum, Thelephora* and *Paxillus* (Colpaert & Van Assche, 1992; Colpaert et al., 2000; Krznaric et al., 2009; Ray et al., 2005; Tam, 1995). Nonetheless, in some cases Cd effects are only evident at higher concentrations, such as 50 mg L^−1^ verified in *Amanita muscaria* growing in solid MMN media (Willenborg, Schmitz & Lelley, 1990), although this species is commonly known to have a high Cd tolerance (Colpaert, 2008; Colpaert & Van Assche, 1992). Here the highest LOAEC values for Cd were observed for *H. subsaponaceum* (3 mg L^−1^) and *Scleroderma* sp. (9 mg L^−1^), both basidiomycetes frequently found on highly polluted soils in the environment (Colpaert, 2008).

The low LOAEC value for *H. crustuliniforme* might be interpreted as a high sensitivity to Cd, however the Tolerance Index (TI%) clearly showed that this species had the most gradual decline in biomass of all Cd treated fungi, indicating less sensitivity to elevated Cd concentrations. For instance, at 9 mg L^−1^ Cd or more, *H. crustuliniforme* was the only species with a TI equal or higher than 20%. This fact emphasizes the importance of using more than one index for interpretations of toxicity data.

Unlike Cd, the range of Zn toxic concentrations is highly variable (generally from 10 to 500 mg L^−1^) depending on the species, strains, or even the type of growth media (Colpaert & Van Assche, 1992; Tam, 1995). Blaudez et al. (2000) verified Zn toxicity on *Suillus luteus* in solid MMN media at a concentration of 25 mg L^−1^, while for the same species Colpaert et al. (2000) found toxicity only at 300 mg L^−1^, but using a different growth media (solid Fries). In an experiment with ECM fungi *in vitro*, Cd^2+^ and Zn^2+^ were also considered the most toxic metals compared to Pb^2+^ and Sb^3−^ (Hartley, Cairney & Meharg, 1997). Nonetheless, Hoiland (1995), who also tested metal toxicity in Basidiomycota, found Cd to be very toxic, but Zn only moderately toxic. Most of the species in the current study presented considerable growth at 1 mg L^−1^ Zn; however, *H. crustuliniforme* had an unexpected reduction on the tolerance index, suggesting that its growth may have been influenced by other factors, such as the media itself. MMN medium usually offers effective results for ECM fungi tests, however some species display different responses to growth media depending on aspects such as nutrient composition or pH (Islam & Ohga, 2013). For example, Willenborg, Schmitz & Lelley (1990) also found poor development of *H. crustuliniforme* in MMN media, which was almost half the growth reached by the same strain in malt extract media.

High metal concentrations exert several toxic effects in fungi and may affect almost all aspects of their metabolism and differentiation, with the cellular membrane being the initial point of action of toxicity if there is a direct contact between the metal and the cellular components (Gadd, 1993), other common effects are the inhibition of enzymes, disruption of membranes, and growth inhibition (Gadd et al., 2012). Exposure to Cd^2+^ resulted in the collapse of mitochondrial membranes in yeasts (Wang et al., 2017).

Several mechanisms of tolerance may act on alleviating metal stresses in fungi, such as increasing metal efflux; reduction of uptake, metal chelation and intracellular sequestration, Ramesh et al. (2009) identified two metallothionein genes in *H. cylindrosporum* capable of restoring the growth of transformed yeasts under Cd toxicity. Cell wall adsorption has also an important contribution in conferring tolerance, especially in the case of Cd (Bellion et al., 2006; Frey, Zierold & Brunner, 2000; Galli, Schüepp & Brunold, 1994). Sequestration into cytosolic vesicles has been shown to be a possible mechanism for Zn tolerance in *H. cylindrosporum* under sub-toxic concentrations (27 mg L^−1^ ZnCl_2_), representing the main pool of free Zn ions in this species (Blaudez & Chalot, 2011).

Yet when exposed to solutions containing high concentrations of metals, such as in this experiment, binding sites in cell walls can be quickly saturated and become an inefficient strategy in preventing toxicity (Colpaert et al., 2011). A study in *Lentiluna edodes* showed high accumulation of Cd in mycelia after only 24 h of exposure in liquid medium (Zhao et al., 2015). Therefore, the physical state of the growth media may have also been responsible for the high Cd sensitivity found in these ECM fungi. Willenborg, Schmitz & Lelley (1990), for instance, verified Cd toxicity in *H. crustuliniforme* only at 50 mg L^−1^, but using solid MMN media, while in our study, with liquid MMN solutions, this species suffered toxicity at 1 mg L^−1^.

When Cd and Zn were added together, the concentrations of 30 and 9 mg L^−1^ Zn resulted in biomass increase in *H. subsaponaceum* and *Scleroderma* sp., respectively, exposed to the highest Cd concentration (9 mg L^−1^). However the Tolerance Index (a percentage of the control biomass) was lower or the same for all Zn treatments in both species (around 80% less, compared to the control—Table S1). This means that although some Zn concentrations promoted fungal growth, they were not able to effectively alleviate Cd toxicity, which suggests that these metals are not sharing the same uptake pathways entirely and/or not competing for the same bonding sites in fungal tissues. However, Cd and Zn toxicity varies depending on the tolerance capacity of different species and strains (Colpaert & Van Assche, 1992); thus, another explanation for the lack of a pronounced Zn ameliorating effect is that all strains used in this assay were highly sensitive to both metals added to the media, considering they were all originated from non-contaminated land.

Despite causing negative effects in certain concentrations, Zn can also be beneficial by acting antagonistically against Cd toxicity in some ECM fungi. Krznaric et al. (2010) reported that tolerance to Cd increased significantly due to Zn additions (80–325 mg L^−1^) in a *S. luteus* strain isolated from contaminated soil. Similar ameliorating effects were observed in other ECM fungi isolates from non-polluted areas by Hartley, Cairney & Meharg (1997); however, a synergistic toxic effect between Cd and Zn was also described by the authors in *S. granulatus*, showing that the interactions between these metals in ectomycorrhizal fungi may occur differently inter or intra-specifically. Even ECM strains originally from polluted areas, which are regarded as more tolerant to toxicity, can suffer from combined effects of Cd and Zn toxicity (Krznaric et al., 2010).

Zn addition led to a few ameliorating effects in both species, mostly at concentrations up to 30 mg L^−1^, however, most treatments were either unaffected by Zn, or caused toxicity in conjunction with Cd, especially at 120 mg L^−1^. It is believed that Zn tolerance mechanisms may increase Cd tolerance when both metals are in excess (Krznaric et al., 2010); thus, if Zn tolerance is not a present trait in the ectomycorrhizal species, it is most likely that the two metals will cause synergistic toxicity instead of alleviating adverse effects. Such results support the affirmation that the toxic effects from multiple metals cannot be predicted from their individual toxicity, as the interactions between them influence their relative toxicity to ECM fungi (Hartley, Cairney & Meharg, 1997). Moreover, tolerance and detoxification of Zn and Cd can happen via different mechanisms. In *Pisolithus tinctorius*, Zn tolerance was conferred by binding the metal to extrahyphal slime (Tam, 1995), while for Cd, vacuole compartmentation and cell wall binding were considered the main metal-detoxification mechanisms in *Paxillus involutus* (Blaudez, Botton & Chalot, 2000). Further investigations are still necessary to elucidate the mechanisms responsible for a possible antagonistic effect.

The fact that radial growth decreased in *H. subsaponaceum* when exposed to high Zn concentrations, but its dry weight did not differ, indicates an increase in mycelial density, which is regarded as an important mechanism to withstand metal toxicity (Hartley et al., 1997). Such mechanism was not observed in *Scleroderma* sp. growing in solid medium, wherein radial growth was unaffected or sometimes increased in response to toxic concentrations. Although this is just one of several mechanisms governing Cd and Zn tolerance in ECM fungi, it is believed that higher density under metal stress is likely to be a significant trait in polluted soils, also affecting the degree of exposure of the plant symbiont (Colpaert et al., 2000). Furthermore, it highlights the importance of using both endpoints (dry weight and radial growth) when screening ECM fungi for metal tolerance.

As suggested earlier, the physical state of growth media can provide different results in terms of toxicity assessment. An advantage of using liquid media, is that it allows a more accurate regulation of the metal concentrations to which the organisms are exposed and it does not depend on growth form (Hartley et al., 1997). However, screenings on solid media allows the assessment of both biomass and radial growth, which can provide more information regarding tolerance aspects, such as the increase in mycelial density observed here in *H. subsaponaceum*. In addition, solid media are more likely to reflect mycelial growth in soils; for instance, basidiomycetes do not completely differentiate in liquid substrates, and this may affect their tolerance to metal toxicity (Hartley et al., 1997). Agar media may offer lower metal bioavailability when compared to liquid media, as it is possible that complexation of metals within agar substrate occurs, masking mycelial response to toxicity (Colpaert et al., 2000); however, it is also useful to avoid acute toxicity due the exposure of highly available metals, as found in liquid media. This experiment clearly demonstrated that the patterns in Cd and Zn sensitivity changed between liquid and solid media and both *H. subsaponaceum* and *Scleroderma* sp. presented higher tolerance indices in agar. Similar effects were also reported by (Colpaert et al., 2000). The high availability of Cd^2+^ in liquid media may have been responsible for a rapid saturation of the binding sites in hyphal cell walls, which can be happen within minutes in these cases (Colpaert et al., 2011), leading to an acute Cd toxic effect.

Despite all the implications, the decision of choosing either liquid or solid media is not often addressed in metal toxicity assessments for ECM fungi in the literature. Out of 16 articles on Cd and/or Zn toxicity in ECM fungi in the past three decades, only five used liquid growth media, for which the Cd and Zn concentrations considered toxic were, in average, 2.2 mg L^−1^ and 123 mg L^−1^ (Colpaert & Van Assche, 1987; Courbot et al., 2004; Grazzioti et al., 2001; Hartley et al., 1997; Tam, 1995), while for the ones that utilized solid media, toxic concentrations were notably higher: in average 12 mg L^−1^ for Cd and 309 mg L^−1^ for Zn (Table 1).

## Conclusions

In the present study, all five ECM species (*A. occidentalis, H. cylindrosporum, H. subsaponaceum, H. crustuliniforme* and *Scleroderma* sp.) tested exhibited high metal sensitivity *in vitro* conditions (liquid media), and Cd was at least 10 times more toxic than Zn, which by itself may explain why Zn had no alleviating effects in Cd toxicity. *H. subsaponaceum* and *Scleroderma* sp. were more tolerant to elevated Cd when grown in solid media compared to liquid, although in both cases higher Zn concentrations were detrimental to these species (synergism) with only a few signs of alleviating Cd toxicity (antagonism). Further research on the mechanisms underlying Zn and Cd antagonistic or synergistic interactions is needed. Additionally, Cd and Zn interactions were also affected by the type of media used, leading to different tolerance patterns, which may help explain the hitherto baffling range of previously recorded results.

A great advantage of using solid media in metal toxicity assays is that it allows the measurement of biomass as well as radial growth and, therefore, the mycelia density, which in this case appears to be a mechanism behind the higher tolerance indices found for *H. subsaponaceum* in contrast to *Scleroderma* sp. Overall, mycorrhizal symbiosis with these species could possibly lead to a better fitness of a host plant exposed to Cd or Zn in contaminated soil, and could be interesting candidates for further investigations.

##  Supplemental Information

10.7717/peerj.4478/supp-1 Table S1 Average tolerance index (%) based on the dry weight (DW) of four ectomycorrhizal fungi grown in liquid media containing different combinations of Cd and Zn doses ( *n* = 4)

10.7717/peerj.4478/supp-2 Supplemental Information 1 Three datasets containing the raw data of dry weight and radial growth of different ectomycorrhizal species under Cd and Zn stress Sheet 1—Dry weight of five ectomycorrhizal species exposed to a range of Cd and Zn concentrations (Data used for Figs. 1 and 2).
Sheet 2—Dry weight of four ECM species exposed to a combination of different Cd and Zn concentrations (Data used for Fig. 3).
Sheet 3—Dry weight and radial growth of *Hebeloma subsaponaceum* and *Scleroderma* sp exposed to a combination of Cd and Zn concentrations (Liquid media vs Solid media) (Data used for Table 2 and Fig. 4).

## Additional Information and Declarations

### Data Availability

The following information was supplied regarding data availability: The raw data are provided in the Supplemental File.
