# Supplementary material for: Cd and Zn interactions and toxicity in ectomycorrhizal basidiomycetes in axenic culture
Source: PeerJ. 2018 Mar 7;6:e4478. doi: 10.7717/peerj.4478 (PMC5845391; doi:10.7717/peerj.4478)
Supplement: Table S1 [file peerj-06-4478-s001.docx]

| Table S1: Average tolerance index (%) based on the dry weight (DW) of four ectomycorrhizal fungi grown in liquid media containing different combinations of Cd and Zn doses (n = 4). | | | | | | | | | | | | | | | | |
| --- | --- | --- | --- | --- | --- | --- | --- | --- | --- | --- | --- | --- | --- | --- | --- | --- |
|  | | *Austroboletus occidentalis* | | | | *Hebeloma crustuliniforme* | | | | *Hebeloma subsaponaceum* | | | | *Scleroderma* sp*.* | | |
| Zn  (mg L^-1^) | | -------------------------------------------------- Cd (mg L^-1^) --------------------------------------------------------- | | | | | | | | | | | | | | |
|  | 0 | | 1 | 9 | 0 | | 1 | 9 | 0 | | 1 | 9 | 0 | | 1 | 9 |
| 0 | 100 | | 20 | 16 | 100 | | 91 | 84 | 100 | | 27 | 9 | 100 | | 85 | 19 |
| 1 | 90 | | 14 | 16 | 104 | | 84 | 82 | 129 | | 28 | 10 | 94 | | 89 | 20 |
| 9 | 74 | | 16 | 14 | 95 | | 86 | 84 | 131 | | 20 | 11 | 155 | | 89 | 23 |
| 30 | 62 | | 18 | 16 | 63 | | 77 | 69 | 136 | | 36 | 13 | 139 | | 99 | 22 |
| TI(%) = (DW-treated / DW-control) x 100 | | | | | | | | | | | | | | | | |
